# Ritodrine-induced rhabdomyolysis and psychiatric symptoms: a case report and literature review

**DOI:** 10.1186/s12884-022-05299-2

**Published:** 2023-01-07

**Authors:** Li Sun, Mimi Tang, Mei Peng, Ping Xu, Ying Wang

**Affiliations:** 1 Department of Pharmacy, the Second Xiangya Hospital of Central South University, Changsha, 410011 China; 2 Institute of Clinical Pharmacy, Central South University, Changsha, 410011 China; 3 Department of Pharmacy, Hunan Provincial Maternal and Child Health Care Hospital, Changsha, 410008 China; 4 Department of Pharmacy, Xiangya Hospital, Central South University, Changsha, 410008 China; 5 Department of obstetrics, the Second Xiangya Hospital of Central South University, Changsha, 410011 China

**Keywords:** Ritodrine, Rhabdomyolysis, Psychiatric symptoms, Pregnancy, Case report

## Abstract

**Background:**

Ritodrine hydrochloride, a β2-adrenergic agonist, has been widely used in Asia and Europe to treat preterm labor in pregnant women. It has some typical side effects, such as palpitations, pulmonary edema, and hypokalemia. Here, we report a case of rhabdomyolysis and psychiatric symptoms might be associated with intravenous ritodrine.

**Case presentation:**

A 32-year-old Chinese primigravida woman who was pregnant with twins by in vitro fertilization-embryo transfer was diagnosed with placenta previa and threatened abortion at 21 gestational weeks (GW). The patient was then treated with ritodrine hydrochloride. The initial dose of ritodrine was 150 μg/min, gradually increasing to 360 μg/min at 23^5/7^ GW and 400 μg/min at 27^1/7^ GW. Magnesium sulfate was added to the ritodrine regimen at 21^5/7^ GW in dosage of 1-2 g/h. Psychiatric symptoms appeared at 24^5/7^, 26^5/7^, and 27^3/7^ GW, manifesting as depression, anxiety, and suicidal tendencies. Severe muscle pain in her limbs and general weakness appeared after six weeks of ritodrine administration, which might have been a sign of rhabdomyolysis resulting from ritodrine administration. After ceasing the administration of ritodrine, the muscle pain and relevant data from laboratory tests on the patient were significantly improved, and her mood was stable. It is worth noting that this is the first time to report psychiatric symptoms may associated with the administration of ritodrine. In addition, we reviewed and analyzed six reported cases of rhabdomyolysis caused by ritodrine.

**Conclusion:**

Our results suggest that we should pay more attention to the risk of rhabdomyolysis and psychiatric symptoms induced by intravenous ritodrine hydrochloride, especially in patients with a history of neuromuscular disorder, or concomitant use of magnesium sulfate.

## Background

The use of β2-adrenergic agonists, such as ritodrine hydrochloride, is common for preventing preterm birth worldwide [1]. Ritodrine is the only agent approved by the US Food and Drug Administration (FDA) for reducing preterm birth within 48 hours of initiation of treatment [2]. In 2012, the American College of Gynecologists (ACOG) recommended β-adrenergic agonists as the first-line tocolytic treatment [3]. However, its use has decreased in some developed countries due to its various side effects, including palpitations, pulmonary edema, hypokalemia, and granulocytopenia [2, 4]. The FDA and the European Medicines Agency (EMA) have recommended discontinuation of oral administration of ritodrine [5]. since the most important objective of tocolysis is to gain time for antenatal corticosteroids to become effective [6]. Moreover, tocolytic agents may be associated with an increased risk of chorioamnionitis [7], and the cardiovascular risk is raised when ritodrine is used for a prolonged period (more than 48 hours) [8]. The product information of ritodrine recommend that long-term (more than 48 hours) ritodrine treatment should closely monitor the risk of cardiovascular-related adverse reactions in pregnant women and fetuses. However, in some other countries, such as China, Japan, and Korea, ritodrine is still used as the first-line tocolytic treatment and is used for a long duration [9, 10].

Here, we report a pregnant woman with twins after being treated with ritodrine for tocolysis for three weeks, who developed psychiatric symptoms manifesting as depression, anxiety, and suicidal tendencies. In addition, she developed rhabdomyolysis after administration of ritodrine for six weeks. It is worth noting that this is the first time to report psychiatric symptoms may associated with the administration of ritodrine. We also review and analyze the reported cases of rhabdomyolysis caused by ritodrine.

## Case presentation

The patient was a 32-year-old Chinese primigravida woman who was pregnant with twins by in vitro fertilization-embryo transfer (IVF-ET). She had no history of neuromuscular disease and was hospitalized three times due to vaginal bleeding from 15^1/7^ GW to 20^6/7^ GW. The agents used during hospitalization were unknown. She was then transferred to The Second Xiangya Hospital of Central South University due to placenta previa and vaginal bleeding at 21 GW. On admission, ritodrine was given by IV for tocolysis. The initial dose of ritodrine was 150 μg/min, gradually increasing to 360 μg/min at 23^5/7^ GW and 400 μg/min at 27^1/7^ GW. Since ritodrine alone could not effectively inhibit contractions, at 21^5/7^ GW, magnesium sulfate (MgSO_4_) was used in combination with the ritodrine regimen to inhibit contractions. Two 12-mg doses of betamethasone were given intramuscularly 24 hours apart at 26^1/7^ GW to accelerate fetal lung maturation. The patient experienced mood swings at 24^5/7^ GW. She felt upset, had an emotional breakdown, and showed suicidal tendencies. She developed anxiety due to poor sleep at 26^5/7^ GW.

At 27^3/7^ GW, she had slept poorly at night and was depressed. She complained of severe muscle pain in the upper and lower limbs and general weakness, accompanied by an abnormal blood myoglobin value of 97.4 μg/L (normal, ≤70 μg/L) and a serum high-sensitivity troponin value of 21.4 pg/mL (normal, 0–14 pg/mL). Her alanine transaminase (ALT) value of 50.8 U/L (normal 7–40 U/L) also began to increase. Her blood creatine kinase (CK) value of 103 U/L (normal, 40–200 U/L) was normal, and Aspartate transaminase (AST) value of 25.6 U/L (normal, 13–35 U/L) was normal. We speculate that rhabdomyolysis had resulted from ritodrine and stopped ritodrine use immediately at 9:00 a.m., while MgSO_4_ at a dose of 1–2 g/h was still used. At 5:00 p.m., the patient stated that the muscle pain was significantly improved, and the mood became stable.

At 27^4/7^ GW, because of severe abdominal pain and uterine dilatation, she delivered two babies. The first female infant weighed 880 g, with an Apgar score of 3/6/7 (1/5/10 minutes), was admitted to the neonatal intensive care unit. The second male infant was delivered by breech extraction and weighed 1080 g, with an Apgar score of 2/2/2 (1/5/10 minutes), did not breathe spontaneously and did not respond to external stimuli. His parents then decided to give up on further rescue. The patient complained of grief during the first day after delivery, and her mood was stable. The serum high-sensitivity troponin T value of ≤8 pg/mL (normal, ≤15.91 pg/mL) and the ALT value of 34.4 U/L (normal, 7–40 U/L) were normal after 2 days postpartum. She was discharged 5 days after delivery, and her postpartum mood was stable. The patient was satisfied with the treatment she received.

## Discussion and conclusions

### Ritodrine-induced rhabdomyolysis

Rhabdomyolysis is a clinical syndrome characterized by skeletal muscle destruction and the release of intracellular muscle content, which can cause systemic complications. The syndrome leads to muscle pain, weakness, dark tea-colored urine, and a significant elevation of serum CK levels. There is no universal definition of rhabdomyolysis, although the common definition includes up-regulated CK levels of more than five times the normal value [11]. In this case, we thought there were three reasons why the patient’s rhabdomyolysis was probably induced by the administration of ritodrine. First, after administration of ritodrine for six weeks, the patient showed symptoms of muscle pain and generalized weakness, and the blood myoglobin, troponin, and ALT values increased. Second, the product information points out that ritodrine has an adverse reaction of rhabdomyolysis. Third, and most importantly, the muscle pain and general weakness were significantly relieved when the patient stopped administration of ritodrine for eight hours. The twins were delivered on the second day after stopping ritodrine. The serum high-sensitivity troponin T value and ALT value was normal after 2 days of postpartum. Due to the timely detection and treatment of this case, no further physical injury occurred. CK is the most sensitive test for indicating damage to muscle cells. Generally, CK increases within 12 hours after muscle damage, reaches a peak after 1–3 days, and begins to decline after 3–5 days [12]. The CK values of this patient did not increase. The possible reason was that the test time was at an early stage, so muscle damage had just occurred, and the serum CK had not yet begun to increase. We tested the CK level when muscle pain appeared in the patient, and it was normal. After that, ritodrine administration was ceased immediately, and no retest was performed after ritodrine was stopped. Therefore, we could not know whether the CK value would have subsequently increased.

In recent years, there have been some reports about the association between rhabdomyolysis and the administration of ritodrine. We searched in the databases of PubMed, Embase, Web of Science, and SinoMed (Chinese biomedical literature service system) using the string “ritodrine AND (rhabdomyolysis OR myalgia OR myodynia OR myosalgia)” as the search strategy from inception to January 2022. After excluding duplicate articles, conference abstracts, and articles in languages other than English and Chinese, six case reports were included in our research [12–17]. The characteristics of these six cases are shown in Table 1.

**Table 1:** Characteristics of the included case reports

First author name and Publication year	Matsuda et al 2002 [13]	Nasu et al 2006 [14]	Verriello et al 2009 [15]	Nakajima et al 2011 [16]	Ogoyama et al 2017 [17]	Zhou et al 2020 [12] (In Chinese)
Country	Japan	Japan	Italy	Japan	Japan	Chinese
Characteristic of pregnant woman	26-year-old Japanese gravida 3, para 0 Monochorionic-diamniotic twins	32-year-old primigravida Singleton pregnancy	31-year-old Caucasian Primigravida Singleton pregnancy	30-year-old Japanese Primigravida Singleton pregnancy	35-year-old Primigravida Singleton pregnancy	34-year-old Singleton pregnancy by IVF-ET
Past medical history	No history of significant medical or surgical history, and no history of neuromuscular disease	Maternal congenital myotonic dystrophy	No history of neuromuscular disorders, alcoholism and drug abuse.	No history of surgical, neuromuscular disorder. Had history of taken alprazolam (0.4 mg/day) prior to conception because of dysautonomia.	Had type II diabetes mellitus. Clinically diagnosed as myositis of unknown etiology or an atypical form of polymyositis at 21^6/7^ GW	Endometrial cancer
Why and when use ritodrine	Increasing contractions that every 7 to 8 min at 23^2/7^ GW.	Premature labor at 31 GW	Premature labor at 28 GW	Diagnosed as placenta previa and mild uterine contractions at 23 GW. Diagnosed vaginal bleeding and frequent uterine contractions at 29^3/7^ GW	Regular uterine contractions with cervical length shortening to 17 mm at 29^6/7^ GW	Irregular contractions at 32 GW
Dosage of ritodrine	23^2/7^ GW: IV, 100 μg/min 24^2/7^ GW: IV, 200 μg/min	31 GW: po, 15 mg/day, initially.	28 GW: po, 300 mg/day. 32 GW: IV, 100 μg/min for 2 days. 32^2/7^ GW: IV, 50 μg/min for other 2 days. 32^4/7^ GW: po, 600 mg/d for 1st day. 32^5/7^ GW: po, 800 mg/d for the following 3 days, and then at 300 mg/day.	23 GW: po, 15 mg/day, initially. 29^3/7^ GW: IV, 50 μmg/min.	29^6/7^ GW, IV, 67 μg/min.	32 GW, IV, 100 mg
Drug combination	MgSO_4_	/	/	Stopped use ritodrine and started use MgSO_4_ (1 g/h).	/	Penicillin, dexamethasone
Gestational age on ADR appeared	28^1/7^ GW	31^3/7^ GW	29 GW	29^3/7^ GW	29^6/7^ GW	32 GW
Symptoms	Slight muscle pain in the lower extremities at 28 GW. Muscle pain increased and dyspnea occurred at 28^6/7^ GW	No evidence of any worsening of the myotonic symptoms.	Mild muscle pain at upper and lower limbs and generalized weakness at 29 GW. After 33^1/7^ GW, muscle pain and weakness worsened. The patient became could neither stand, nor walk at 36 GW.	Extreme muscle pain in the upper and lower limbs and general weakness at 29^3/7^ GW.	Severe muscle pain in the limbs appeared after ritodrine administration 3 hours.	Local pain appeared after 9 hours of treatment as long as the front thigh bended, which would disappeared as long as straightening. One hour later, emergency cesarean section was performed due to fetal distress. The muscle pain of lower limbs increased after cesarean section. The urine was slightly darker and yellow.
Laboratory tests	28^1/7^ GW: CK 322 IU/L; AST 86 IU/L; ALT 102 IU/L; 28^6/7^ GW: CK 1167 IU/L, Mb(blood) 280 ng/mL. Mb(urinary) 12 ng/mL. Urinalysis was positive for occult blood and revealed 3–4 red blood cells/field and positive granular casts. SpO2 90%.	CPK 10,897 mg/dl, and myoglobinuria (1800 ng/dl)	36 GW: CK 25000 UI/L; AST 150 UI/L; ALT 140 UI/L. Mb(urinary) 2.3 ng/dl.	Before the IV administration: CK 7200 IU/L; CK-MB 208 IU/L; AST 163 IU/L; ALT 74 IU/L; LDH 536 IU/L. The next day of the IV administration, CK 87300 IU/L; CK-MB 2040 IU/L; AST 1164 IU/L; ALT 248 IU/L; LDH 3380 IU/L; Mb(blood) 11,200 ng/mL; Mb(urinary) 615 ng/mL.	29^6/7^ GW: CPK 32019 U/L. myoglobinuria.	On the first postpartum day, Mb>3838 ng/mL, troponin 0.078 ng/mL, CK 20555 U/L; LDH 1229 U/L, CK-MB 248 U/L; ALT 51.4 IU/L; AST 333 IU/L; Urinalysis was positive for occult blood (3+)
Outcomes of infant	28^6/7^ GW performed an emergency cesarean section. Both infants exhibited respiratory distress syndrome, both did well after extubation with no other problems	Spontaneously delivered a healthy male baby at 37 GW	The woman underwent operative delivery, the baby was normal without peri-partum sufferance.	An emergency cesarean section was carried out at 29^5/7^ GW. The neonate was admitted to the neonatal intensive care unit due to immaturity, and had respiratory distress syndrome, patent ductus arteriosus and ventricular septal defect. The blood CK level was 799 IU/L.	An emergency cesarean section was carried out at 31^6/7^ GW. After birth, the infant was diagnosed with myotonic dystrophy	Emergency cesarean section was performed due to fetal distress after 10 h of administration. The postnatal condition of the fetus was not described.
Outcomes of pregnant woman	On the third postpartum day, maternal oxygenation had improved, and oxygen administration was discontinued. In addition, the muscle pain disappeared, and CK, AST and ALT levels normalized soon after delivery	The laboratory data improved gradually with the serum CPK levels at 955 mg/dl. (Her serum CPK levels at admission was 1919 mg/dl)	In one month, she could stand and walk again without help, but a mild weakness still persisted at girdle muscles. Levels of blood CK were still high (7000 UI/L). Three months later, both neurological assessment and CK levels were normal	the muscle pain disappeared, and CK, AST, ALT, LDH, and blood myoglobin levels normalized soon after delivery. On the eighth postpartum day, the laboratory data improved gradually and the CK levels were at 107 IU/L	The muscle pain soon disappeared and the elevated CPK and myoglobinuria immediately resolved	The muscle pain disappeared after 2 days of postpartum. Mb 1154.6 ng/mL; troponin 0.039 ng/mL; CK 28020 U/L; CK-MB 272 U/L； LDH 1064 U/L; ALT 99.2 IU/L; AST 538 IU/L; Urinalysis was positive for occult blood (3+). The laboratory test was normal after 7 days of postpartum.

*Abbreviation:* *MgSO*_*4*_ magnesium sulfate; *GW* gestational weeks; *CK* creatine kinase or creatinine phosphokinase (CPK);* CK-MB*: MB isoenzyme of creatine kinase; *AST* Aspartate transaminase; *ALT* alanine transaminase; *LDH* lactate dehydrogenase; *Mb* myoglobin; *IV* intravenous; *IVF-ET* in vitro fertilization-embryo transfer; *po* oral

In the cases reported by Nasu et al. [14] and Ogoyama et al. [17], two pregnant women were previously diagnosed with maternal congenital myotonic dystrophy and atypical polymyositis, respectively, because of which rhabdomyolysis occurred relatively early in these women after ritodrine treatment. Nasu et al. [14] also reported a pregnant woman who received only oral ritodrine hydrochloride at a dose of 15 mg/day. Three days after administration, serum CK levels were markedly elevated and myoglobinuria was detected. Ogoyama et al. [17] reported a pregnant woman who was administered IV ritodrine at a low dosage of 67 μg/min. After three hours of ritodrine administration, severe muscle pain developed and CK levels were elevated with myoglobinuria. These cases suggest that patients with a history of neuromuscular disorders are prone to rhabdomyolysis adverse reactions when using ritodrine, and the occurrence time is relatively early and progresses relatively quickly. Ogoyama et al. [17] also found that among most patients with myotonic dystrophy, ritodrine-induced rhabdomyolysis occurred within a few hours to one day after commencing IV ritodrine, whereas in patients without myotonic dystrophy, rhabdomyolysis occurred after several days or weeks. The patient we reported here had no history of neuromuscular disorder, and the appearance of symptoms of muscle pain and general weakness occurred six weeks after the administration of ritodrine.

A retrospective cohort study [18] found that in Japan, the proportion of patients who underwent ritodrine treatment for ≥28 days was 28.7%, with only 17.2% receiving treatment for ≤48 hours.The proportional occurrence of maternal adverse effects was significantly higher for women who underwent ritodrine treatment for ≥28 days than ≤48 hours, especially in thromboembolism and gestational diabetes mellitus. The incidence of rhabdomyolysis was 3.8% among the women who underwent tocolytic treatment for ≥28 days, and 8.7% among the women who underwent tocolytic treatmen ≤48. The product information of ritodrine recommend that long-term (more than 48 hours) ritodrine treatment should closely monitor the risk of cardiovascular-related adverse reactions in pregnant women and fetuses. The guidelines [3] recommend that tocolytic treatment should be limited to 48 hours due to the risk of risk of cardiovascular. However, in clinical practice, long-term tocolysis above 48 hours is common in both China and Japan [10, 18]. This prompts us to focus on the risk of adverse drug reactions in patients with long-term ritodrine use.

It is worth mentioning that the woman in our reported case had concomitant use of MgSO_4_. Hypermagnesemia can lead to neuromuscular toxicity, severe hypermagnesemia can result in loss of deep tendon reflexes and muscle paralysis. The plasma Mg concentration of the pregnant woman in this case was normal during hospitalization. Matsuda et al. [19] performed a retrospective cohort study to evaluate the relationship between tocolytic therapy and CK levels. They found that the total doses of ritodrine and MgSO_4_ in the abnormal CK group were significantly higher than in the normal CK group. Furthermore, 100% of the abnormal CK group had concomitant use of MgSO_4_, while 50% of the normal CK group had concomitant use of MgSO_4_. However, this study did not compare the changes in CK levels between ritodrine use alone and ritodrine combined with MgSO_4_. Yada et al. [10] found that the use of ritodrine in combination with MgSO_4_ was associated with the occurrence of critical neonatal hyperkalemia in late preterm infants. Therefore, in pregnant women who use ritodrine and concomitant MgSO_4_, we should pay close attention to the risk of adverse reactions in mothers and newborns.

### Ritodrine-induced psychiatric symptoms

Ritodrine can cause adverse reactions in the central nervous system, including dizziness, lethargy, headache, and tremors [9], but there were no previous reports of psychiatric symptoms. To the best of our knowledge, this is the first case reporting that IV ritodrine probably associated with psychiatric symptoms. We analyzed all the medications that were administered during the patient’s hospitalization, including MgSO_4_, cefoxitin, progesterone, dydrogesterone, potassium chloride, and iron polysaccharide complex capsules. None of these agents was considered capable of inducing the occurrence of psychiatric symptoms. Liu et al. [20] performed a 14-year retrospective study and found that ritodrine treatment for preterm labor was a significant risk factor for postpartum depression, especially in the form of an injection. This study lets us link the psychiatric symptoms of the patient in the present study with ritodrine. According to the patient’s self-report, she had no history of psychiatric disorders but used to be rebellious in adolescence and even had suicidal tendencies once. However, she did not seek medical treatment at that time and had shown great emotional stability over the years. The patient was admitted at 21 GW and began IV ritodrine. Psychiatric symptoms appeared at 24^5/7^ GW, 26^5/7^ GW, and 27^3/7^ GW, manifesting as depression, anxiety, and suicidal tendencies. The patient complained of severe muscle pain in the upper and lower limbs at 27^3/7^ GW. We immediately ceased administration of ritodrine at 9:00 a.m. The elimination half-life of ritodrine is 1.7–2.6 hours. The drug is eliminated after five half-lives (8.5–13 hours). The patient’s psychiatric symptoms significantly improved, and her mental state was relatively stable 8 hours after drug withdrawal. At follow-up after discharge, the patient complained of worrying about her daughter, but she was in a stable mood. Therefore, we speculate that the psychiatric symptoms of this patient during pregnancy may be caused by ritodrine.

In summary, we report a case of a woman pregnant with twins who developed rhabdomyolysis and psychiatric symptoms after IV ritodrine hydrochloride. We should be alert to the risk of rhabdomyolysis in patients who use ritodrine for tocolytic treatment, especially in patients with a history of neuromuscular disorder, or concomitant use of MgSO_4_. For pregnant women might have high risk factor of rhabdomyolysis used ritodrine, we should monitor the serum CK levels, urine color, and the appearance of muscle pain. If there are any abnormality, the ritodrine should be ceased and evaluated. Since the association between psychiatric symptoms and ritodrine was first reported by us, further cohort studies with large sample are needed to confirm whether IV ritodrine could induce psychiatric symptoms, and whether the psychiatric symptoms are related to the dosage and duration of ritodrine. Pregnant women at risk for psychiatric disorders who use ritodrine should be regularly monitored with self-rating scale to reduce the risk of developing psychiatric disorders.

## Data Availability

For further details, the corresponding author can be contracted.

## Abbreviations

FDA: Food and Drug Administration
ACOG: American College of Gynecologists
EMA: European Medicines Agency
IVF-ET: in vitro fertilization-embryo transfer
IV: intravenous
GW: gestational weeks
MgSO4: magnesium sulfate
ALT: alanine transaminase
AST: Aspartate transaminase
CK: creatine kinase or creatinine phosphokinase (CPK)
CK-MB: MB isoenzyme of creatine kinase
LDH: lactate dehydrogenase
Mb: myoglobin
po: oral.

## References

[1] Takagi K, Satoh T (2009). Is long-term tocolysis effective for threatened premature labour?. J Int Med Res.

[2] Shinohara S, Sunami R, Uchida Y, Hirata S, Suzuki K (2017). Association between total dose of ritodrine hydrochloride and pulmonary oedema in twin pregnancy: a retrospective cohort study in Japan. BMJ Open.

[3] ACOG (2012). practice bulletin no. 127: management of preterm labor. Obstet Gynecol.

[4] Haas DM, Caldwell DM, Kirkpatrick P, McIntosh JJ, Welton NJ (2012). Tocolytic therapy for preterm delivery: systematic review and network meta-analysis. Bmj..

[5] Shimokawa S, Sakata A, Suga Y, Isoda K, Itai S, Nagase K (2019). Incidence and risk factors of neonatal hypoglycemia after ritodrine therapy in premature labor: a retrospective cohort study. J Pharm Health Care Sci.

[6] Di Renzo GC, Cabero Roura L, Facchinetti F, Helmer H, Hubinont C, Jacobsson B (2017). Preterm labor and birth management: recommendations from the European Association of Perinatal Medicine. J Matern Fetal Neonatal Med.

[7] Siegler Y, Weiner Z, Solt I, ACOG (2020). Practice Bulletin No. 217: Prelabor Rupture of Membranes. Obstet Gynecol.

[8] European Medicines Agency: PRAC recommends restricted use of shortacting beta-agonists in obstetric indications. http://www.ema.europa.eu/docs/en_GB/document_library/Referrals_document/Short-acting_betaagonists/Recommendation_provided_by_Pharmacovigilance_Risk_Assessment_Committee/WC500148669.pdf. .

[9] Kim MK, Lee SM, Oh JW, Kim SY, Jeong HG, Kim SM (2018). Efficacy and side effect of ritodrine and magnesium sulfate in threatened preterm labor. Obstet Gynecol Sci.

[10] Yada Y, Ohkuchi A, Otsuki K, Goishi K, Takahashi M, Yonemoto N (2020). Synergic interaction between ritodrine and magnesium sulfate on the occurrence of critical neonatal hyperkalemia: a Japanese nationwide retrospective cohort study. Sci Rep.

[11] Sawhney JS, Kasotakis G, Goldenberg A, Abramson S, Dodgion C, Patel N, et al. Management of rhabdomyolysis: a practice management guideline from the eastern Association for the Surgery of trauma. Am J Surg. 2021. 10.1016/j.amjsurg.2021.11.022. 10.1016/j.amjsurg.2021.11.022 34836603

[12] Zhou LZY, Wang L, Chen X (2020). Diagnosis and treatment of rhabdomyolysis induced by ritodrine hydrochloride in the third trimester: a cese report and literature review. Chin J Fam Plann.

[13] Matsuda Y, Nagayoshi Y, Kirihara N (2002). Rhabdomyolysis during prolonged intravenous tocolytic therapy. J Perinat Med.

[14] Nasu K, Sugano T, Yoshimatsu J, Narahara H (2006). Rhabdomyolysis caused by tocolysis with oral ritodrine hydrochloride in a pregnant patient with myotonic dystrophy. Gynecol Obstet Investig.

[15] Verriello L, D'Amico D, Pauletto G, Gigli GL, Bergonzi P (2009). Rhabdomyolysis caused by tocolytic therapy with ritodrine hydrochloride. Neuromuscul Disord.

[16] Nakajima Y, Masaoka N, Tsuduki Y, Honda N, Sakai M (2011). Rhabdomyolysis caused by tocolytic therapy with oral ritodrine hydrochloride in a pregnant woman with placenta previa. J Obstet Gynaecol Res.

[17] Ogoyama M, Takahashi H, Kobayashi Y, Usui R, Matsubara S (2017). Ritodrine-induced rhabdomyolysis, infantile myotonic dystrophy, and maternal myotonic dystrophy unveiled. J Obstet Gynaecol Res.

[18] Shigemi D, Aso S, Yasunaga H (2019). Inappropriate use of ritodrine hydrochloride for threatened preterm birth in Japan: a retrospective cohort study using a national inpatient database. BMC Pregnancy Childbirth.

[19] Matsuda Y, Nagayoshi Y, Kirihara N (2002). Evaluation of creatine kinase level during long-term tocolysis. J Perinat Med.

[20] Liu JM, Liu CY, Hsu RJ, Chang FW (2021). Preterm labor using Tocolysis as a possible risk factor for postpartum depression: a 14-year population-based study in Taiwan. Int J Environ Res Public Health.

